# Management of nanomaterials safety in research environment

**DOI:** 10.1186/1743-8977-7-40

**Published:** 2010-12-10

**Authors:** Amela Groso, Alke Petri-Fink, Arnaud Magrez, Michael Riediker, Thierry Meyer

**Affiliations:** 1 Occupational Safety and Health, School of Basic Sciences, Ecole Polytechnique Fédérale de Lausanne Switzerland; 2 Advanced Particles Group, Department of Chemistry, University of Fribourg, Switzerland; 3 Laboratory of Nanostructures and Novel Electronic Materials, Ecole Polytechnique Fédérale de Lausanne, Switzerland; 4 Institute for Work and Health (Institut universitaire romand de Santé au Travail), Lausanne, Switzerland

## Abstract

Despite numerous discussions, workshops, reviews and reports about responsible development of nanotechnology, information describing health and environmental risk of engineered nanoparticles or nanomaterials is severely lacking and thus insufficient for completing rigorous risk assessment on their use. However, since preliminary scientific evaluations indicate that there are reasonable suspicions that activities involving nanomaterials might have damaging effects on human health; the precautionary principle must be applied. Public and private institutions as well as industries have the duty to adopt preventive and protective measures proportionate to the risk intensity and the desired level of protection. In this work, we present a practical, 'user-friendly' procedure for a university-wide safety and health management of nanomaterials, developed as a multi-stakeholder effort (government, accident insurance, researchers and experts for occupational safety and health). The process starts using a schematic decision tree that allows classifying the nano laboratory into three ***hazard ***classes similar to a control banding approach (from *Nano 3 *- highest hazard to *Nano1 *- lowest hazard). Classifying laboratories into ***risk ***classes would require considering actual or potential exposure to the nanomaterial as well as statistical data on health effects of exposure. Due to the fact that these data (as well as exposure limits for each individual material) are not available, risk classes could not be determined. For each hazard level we then provide a list of required risk mitigation measures (technical, organizational and personal). The target 'users' of this safety and health methodology are researchers and safety officers. They can rapidly access the precautionary hazard class of their activities and the corresponding adequate safety and health measures. We succeed in convincing scientist dealing with nano-activities that adequate safety measures and management are promoting innovation and discoveries by ensuring them a safe environment even in the case of very novel products. The proposed measures are not considered as constraints but as a support to their research. This methodology is being implemented at the Ecole Polytechnique de Lausanne in over 100 research labs dealing with nanomaterials. It is our opinion that it would be useful to other research and academia institutions as well.

## Introduction

In the last years, nanotechnology has become a key word of public interest, since it brings together different areas of science and benefits from an interdisciplinary or "converging" approach and is expected to lead to innovations that can contribute to addressing many of the problems facing today's society. A scientific and technical revolution has begun that is based upon the ability to systematically organize and manipulate matter on the nanometer length scale. Several nanotechnology-based products have been marketed including electronic components, scratch-free paint, sports equipment, wrinkle- and stain-resistant fabrics, sun creams, and medical products (e.g. bandages, heart valves, MRI contrast agents). Analysts estimate that the market for such products is currently around hundred of billions of euro and could rise to one trillion by 2015 [1]. Accordingly, potential occupational and public exposure to manufactured nanoparticles will increase dramatically in the near future. Many researchers have addressed the toxicity issues associated with different nanoparticles in vitro and in vivo [2-4]. However, information describing the relative health and environmental risk assessment of engineered nanoparticles or nanomaterials (hereafter, we will use ISO/TR 12885 definition [5] of engineered nanomaterials) is severely lacking. Effects of nanoparticle properties on the immune system are still being explored, and studies of many nanoparticle preparations generally fall into two categories: (a) responses to nanoparticles that are specifically modified to stimulate the immune system or (b) undesirable side-effects of nanoparticles [6]. One initiative that tried to shed light on this issue is a recently completed global review of completed and nearly completed environment, health and safety research on nanomaterials and nanotechnology [7]. The resulting EMERGNANO report is a unique attempt to identify and assess worldwide progress in relation to nanotechnology risk issues. There is no doubt a consensus among producers and users that there is "a need for better characterization of nanotechnology constructs" and for the production of "reagent-grade" nanomaterials, which permit comparison between researches and tests [8]. Recently, this lack of progress in nanotoxicology came under the spotlight [9] again when researchers reported that nanoparticles had been found in the lungs of seven women who had become ill after working in a paint factory in China; two of them later died [10]. However, it remains unclear if the illnesses were caused by the nanoparticles or other chemicals [11]. There is also a widespread agreement [12] that this tragic accident could have been prevented by proper health and safety procedures - the women only occasionally wore masks and the first symptoms appeared five months after the ventilation unit in the factory broke down. At the very least what happened in China emphasises the importance of proper risk management when workers are exposed to nanoparticles for prolonged periods [9].

Initial safety and health strategies [13-15] were analogies to those for chemicals and powders. Yet, they are not applied consistently and users and producers seem to rely mostly on personal protective equipment [16]. More recent efforts aim at developing strategies that target nano-specific aspects [17-20]. Recently, the Swiss Government published a precautionary matrix that allows an initial assessment of the risks of nanomaterial applications without requiring detailed knowledge on the toxicology of the nanomaterials involved [21]. Such preliminary information is essential for simplified, so-called control-banding approaches that group risks in broad classes and then define different levels (or bands) of protection efforts [22].

A professional **hazard **is any potential source of damage, injury or harmful effect in respect of a thing or person in certain conditions of the workplace. A hazard is a characteristics of something (tool, machine, product, but also instruction, activity, organization etc..) that can negatively affect the integrity of a person or thing.

**Risks **are associated with the nature of material and exposures that people have to that material. For a full risk assessment, detailed information is required about the material (chemical composition of nanoparticles, Material Safety Data Sheet when available, particle morphology, aspect ratio, particle size distribution, zeta potential, solubility, known hazards) as well as about the full process descriptions where nanomaterials are used or produced. For each uptake route (respiratory organs, skin, gut) type and level of exposure need to be investigated for each process step. This is a challenge, because it is unclear which characteristics drive the toxicity of nanomaterials and thus need to be measured. Several methods already exist to measure nanoparticle concentrations in air. Mobility particle sizers usually provide reliable and comparable data [23]. However, they are large, expensive and require extensive training, inducing hindrances for routine exposure assessment of workers or researchers.

All this complicates risk assessment considerably. Nevertheless, given the lack of current knowledge about the toxicity of nanomaterials, the difficulty to compare the results obtained from various investigations, and the concern that the bulk materials' safety data sheets might not adequately reflect the real hazardousness of nanomaterials, precaution recommends that all nanomaterials shall be considered potentially hazardous unless sufficient information to the contrary is obtained.

### Precautionary principle

At the Rio Conference on the Environment and development in 1992, world leaders agreed on the Precautionary principle stated in the following terms: 'In order to protect the environment, the precautionary principle shall be widely applied by states according to their capability. Where there are threats of serious or irreversible damage, lack of full scientific certainty shall not be a reason for postponing cost-effective measures to prevent environmental degradation' [24]. While this principle has primarily been used internationally around environmental health issues, other groups are adopting this philosophy to protect the health of workers. In 1996, the American Public Health Association passed a resolution entitled, "The Precautionary Principle and Chemical Exposure Standards for the Workplace". This resolution recognized the need for implementing the precautionary approach, where chemicals are considered potentially dangerous, until the extent of its toxicity is sufficiently known, and the establishment of strict, preventive chemical exposure limits. In February 2000, the European Commission published a Commission Communication on the precautionary principle (EU Resolution on the Precautionary Principle, 2000) providing a general framework for its use in EU policy [25].

So, if a preliminary scientific evaluation emphasizes that there are reasonable grounds for concern that a particular activity might lead to damaging effects on the environment, or on human, animal or plant health, the precautionary principle is triggered. Within this context, we consider the precautionary principle as directly applicable to emerging nanotechnologies.

Being responsible for safety and health in an research institution, we had to determine which actions should be taken, potential effects of taking no actions, the uncertainties inherent in the scientific evaluation, and the views on how to manage the risks. The adopted measures had to be proportionate to the level of risk and the desired level of protection and will evolve with forthcoming knowledge. It is possible that level of protection may be eased, especially for Nano 3 as defined here, as more is known about specific nanomaterials.

### Objectives and motivation

**Figure 1:**
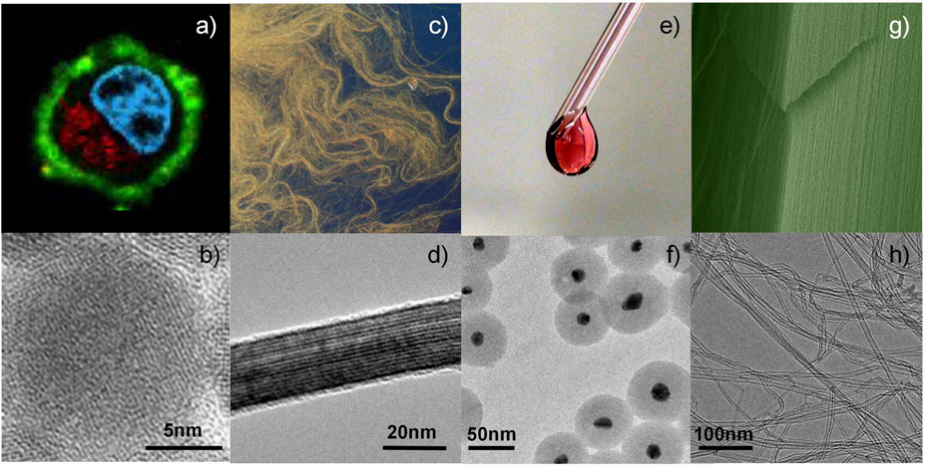
**Different forms of nanomaterials produced at the EPFL**. Examples of different forms of nanomaterials produced at the EPFL. a) Confocal micrograph of cells exposed to Cy3.5 labelled nanoparticles (red). b) High resolution transmission electron micrograph of superparamagnetic iron oxide nanoparticles. c) Optical micrograph of V_2_O_5 _nanowires [47]. d) Transmission electron micrograph of V_2_O_5 _nanowires. e) Suspension of gold nanoparticles. f) Transmission electron micrograph of gold/silica core-shell nanoparticles. g) Scanning Electron Micrograph of aligned carbon nanotubes forest [48] h) Transmission Electron Micrograph of carbon nanotubes produced by Chemical Vapour Deposition.

At EPFL (Ecole Polytechnique Fédérale de Lausanne), over 30 research groups (in basic sciences, engineering or life sciences) produce, modify or use engineered nanomaterials (Figure 1) in approximately 100 laboratories with over 300 different associated production or characterization processes. Classical risk assessment methods (Hazard and Operability Studies (HAZOP) - often used to analyze risks in chemical processes, Failure Mode and Effects Analysis (FMEA) - often used in industry to evaluate the effects of potential failure modes, etc.) would require around 2000 man/day workload - huge resources, nearly impossible to obtain. Some other institutions have already developed best practices guides and safety management procedures for nanomaterials [26-29]. However, they mainly propose a risk analysis approach for each individual process and particle type, which is not very practical for large research centers with many different, constantly changing forms of nano-related studies and laboratories. Or alternatively, good laboratory practices are proposed [30,31] that apparently are not well respected. Published in the February issue of Nature Nanotechnology [32], Jesus Santamaria and his team have conducted an online survey to identify what safety practices researchers are following in their laboratories. The responses of the 240 participants shed some light on what is going on. The questions covered: details of the materials and processing methods used; safety measures; waste disposal procedure, and knowledge of legislation for handling nanomaterials. One of the most surprising results [33] is that nearly three quarters of respondents reported not having internal rules to follow regarding the handling nanomaterials (approximately half of them didn't have rules and over a quarter were not aware of any internal regulations). All this led to the development of a methodology and procedure helping to answer questions related to safety and health for present and future users of nanomaterials in university setting. The methodology was introduced and tested for applicability at the EPFL.

### Development of safety procedure for nanoobjects production/use

A Nanosafe team consisting of three safety and health specialists, one nano-health and occupational hygiene expert, one insurance representative, three EPFL scientists and nanoparticles' users (production and use) and one representative of State Secretariat for Economic Affairs was appointed to develop a procedure for managing the occupational safety and health risks relevant to research laboratories producing and using nanomaterials. The procedure consists of two parts. Using a decision tree we sort the "nano-laboratories" into three hazard classes (Nano 3 = highest hazard to Nano 1 = lowest hazard), which corresponds to analogue approaches applied to other hazard types (biohazard, radioprotection or chemistry). We then provide a list of required prevention/protection measures (safety barriers) for each hazard level. The target users of this safety and health methodology are at first researchers. They can rapidly access the hazard class of their activity and the corresponding adequate safety and health measures. More detailed analysis of specific activities can be undertaken by safety and health experts when needed. According to our opinion and experience, the proposed management of nanomaterial safety is not stifling or harming innovation, as it is sometimes feared among researchers [34].

### Decision tree for laboratory type determination

**Figure 2:**
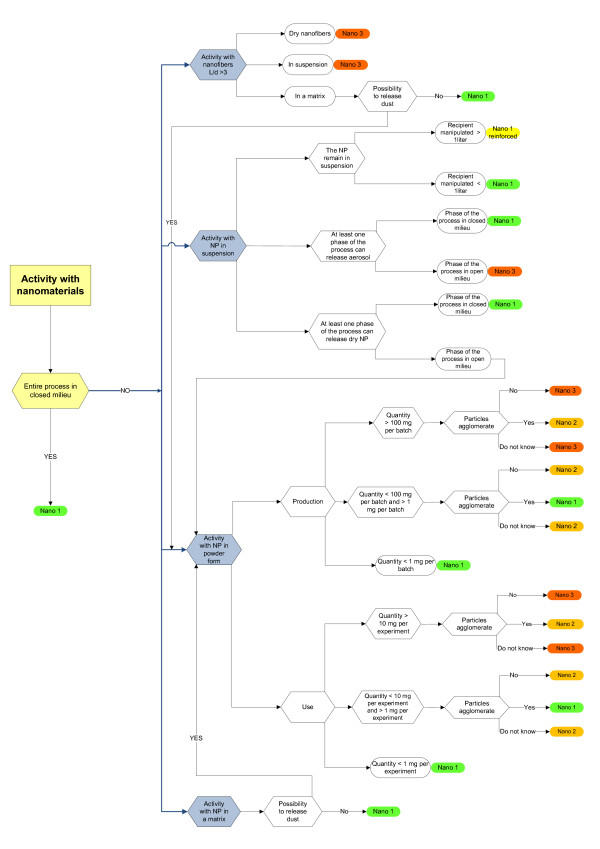
**Decisions tree used for determination of Nano hazard type**. Questions to be answered by nanomaterials users and producers when determining laboratory (Nano hazard) type. NP: nanoparticles, L/d: length - diameter aspect ratio.

Figure 2 depicts the questions to be answered by nanomaterial users and producers (only research environment is considered, industrial processes are not discussed) when classifying their activities. Exposure to nanomaterials may happen by ingestion, inhalation, injection and dermal contact. The main occupational exposure routes are the respiration tract and the skin. Consequently, the first differentiation regards the environment, whether the process is carried out in a closed (complete process confinement) or open system. In case the process is not fully enclosed (glove box or completely sealed environment), different types of activities with nanomaterials are subsequently discussed individually.

#### Activity with nanofibers

The scientific community is mostly concerned about the toxicity/carcinogenicity of manufactured nanofibers (nanomaterials with length-diameter aspect ratio larger than 3) because of their morphological resemblance to asbestos. Inhalation of asbestos fibers is known to induce asbestosis (a progressive fibrotic disease of the lung), lung and pleura cancer. The health hazards of nanofibers are mostly limited to carbon nanotubes and are the subject of an intensive research. Results of already established toxicity studies show a clear morphology-toxicity relationship for carbon nanotubes, [35,36] as previously observed for asbestos fibers [37]. However, synthesis of nanofibers is being continuously under progress and, as a result, nanofibers can be made out of nearly any material nowadays. Some of them will very likely resemble more closely to asbestos than carbon nanotubes by their size, chemical composition or surface properties. They open the possibility of making nanofibers with undesired harmfulness [38], which could be putatively equal or even higher than the one of asbestos. Hence, all activities, either with dry nanofibers or nanofibers in suspension will situate the laboratory in the *Nano 3 *category (Figure 2) except for those where nanofibers are strongly interacting with the matrix (composites), preventing the materials to be released in the environment (refer to activities with nanoobjects in solid matrix).

#### Activity with nanoobjects in powder

The exposure dose is a function of exposure level and duration of exposure [39]. In traditional risk assessment, exposure doses are compared to Threshold Limit Values (TLV). In Switzerland, there are no TLV that were specifically generated for nanomaterials but there are TLV [40] for diesel particles (0.1 mg/m^3^), and fumed silica (4 mg/m^3^). The British Standards Institute proposed, as a pragmatic guidance, the following [14]: if a material is classified in its larger form as carcinogenic, mutagenic, asthmagenic or a reproductive toxin and a TLV is known, its nano form will have a TLV 10 times smaller. For insoluble materials, their nanoform will have a larger safety margin (1/15 the non-nanoscale TLV); for soluble materials, it is reduced by a half. These considerations are included in our approach.

For nanopowders we distinguished ***production ***and ***handling ***(Figure 2). The lower limit up to which a ***production ***laboratory is classified in *Nano 1 *is set to 1 mg of nanomaterial present at any given moment. If one assumes a volume of 10 cubic-meters in which particles could spread around an equipment or a person in case of incidents, 1 mg corresponds to the TLV for diesel particles (1 mg/10 m^3 ^= 0.1 mg/m^3^). From a practical perspective, 1 mg constitutes the lower detection limit of many common laboratory balances. Laboratories with more than 1 mg but less than 100 mg are classified as *Nano 2 or Nano 3*, depending on the agglomeration status. Nanomaterials exhibiting large surfaces might be toxic or catalyze the production of toxic substances. Furthermore, nanoparticles often display good transfer into [41] and across epithelial cells [42] and then distribute to other body compartments probably as a function of size and surface properties [43]. Thus, laboratories with single particles or unstable agglomerates are in *Nano 3*. Stable agglomerates and aggregates do not have the nano-specific route of transfer ("normal" transports can still occur) and are expected to affect health more like "classical" ambient air pollution particles [44]. Consequently, activities with agglomerates are classified in *Nano 2*.

Laboratories using more than 100 mg (a considerable quantity in a research environment) are always classified as *Nano 3*.

For the hazard classification of nanoparticles ***handling ***activities, an identical approach as for production activities is taken (Figure 2), with the exception that the upper limit for *Nano 3 *is reduced by a factor ten (10 mg). Very often, particles are supplied by other laboratories or external suppliers, where occupational safety and health team cannot control the process as well as for home-made particles. Furthermore, users manipulate such particles more often in confined spaces.

#### Activity with nanoobjects in suspension

Many applications and investigations use nanoparticles in colloidal suspensions. Nanoparticles in suspension are rarely encountered as bare nanoparticles but have their surface modified in order to ensure colloidal stability or subsequent surface derivatization. This increases the complexity when determining colloids toxic action.

The hazards related to nanomaterials suspension is not only influenced by the nature of particles but also by the dispersant. The decision tree (Figure 2) is organized accordingly: For manipulated quantities superior to 1 liter the nature of the used dispersant (flammable, toxic etc.) is considered equally important: working under the fume hood is mandatory in laboratories classified as "reinforced hazard level Nano 1". If particles remain in suspension and the manipulated quantity is smaller than 1 liter, the laboratory is classified Nano 1 (equivalent to a classical chemical lab). If aerosols can be released, the equipment has to be placed in a closed environment. Airborne droplets can carry large amounts of nanoparticles into the lungs, and especially small droplets can dry quickly while the solid parts remain airborne. Laboratories with such processes are thus considered as Nano 3.

#### Activity with nanoobjects in solid matrix

Studying composites with nanoobjects embedded in polymer or in ceramic matrices represents one of the most important activities with nanomaterials at EPFL. The preparation of composites is either treated as "Activity with nanoobjects in suspension" or "Activity with nanoobjects in powder" when performed in solution or in dry conditions, respectively. The laboratory is treated as *Nano 1 *if material characterization and post-preparation processing activities do not include any mechanical or thermal treatment. If dust can be released during the manipulation or if composites are friable, laboratory is treated as "Activity with nanoobjects in powder".

### Protective measures

**Figure 3:**
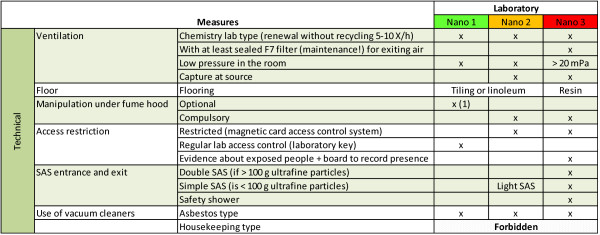
**Technical safety measures**. Technical safety actions applied to laboratory classified 'Nano'. (1) Reinforced type 1 = type 1 plus obligatory manipulation under fume hood.

**Figure 4:**
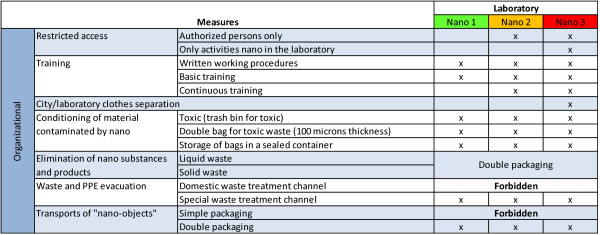
**Organizational safety measures**. Organizational safety actions applied to laboratories classified 'nano'.

**Figure 5:**
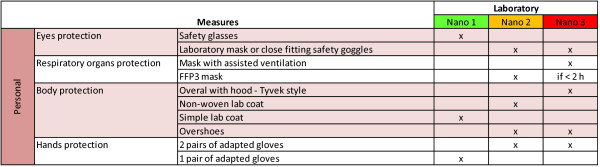
**Personal safety measures**. Personal safety actions applied to laboratories classified 'nano'.

**Figure 6:**
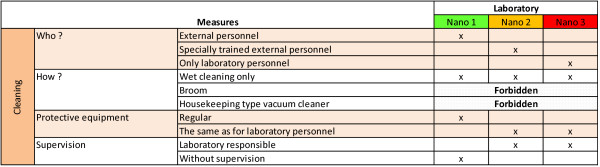
**Cleaning management**. Organization of cleaning for different nano laboratories' types.

Inhalation and skin contact are considered as most important exposure routes. Measures are organized in consequence. Technical, organizational and personal protective measures for different laboratory (Nano hazard) types are presented in Figure 3, 4 and 5. Even though management of cleaning can be considered as a part of organizational measures, it is separated (Figure 6) to underline its importance.

#### Technical measures

As illustrated in Figure 3 laboratories with hazard level *Nano 3 *will require rather extensive technical measures with capture at source, exiting air filtering with at least a F7 filter [EN 779 -European Standard for ventilation filters. F7 has 80-90% average efficiency for 0.4 μm particles], and access restrictions using a security vestibule (double door). A double security vestibule with a safety shower is required for each new lab while simple one can be installed for existing laboratories without sufficient space.

#### Organizational measures

Most organizational protective measures are similar for all laboratory types (see Figure 4). Measures not listed in the figure are the following:

- Each laboratory must have a responsible person (nano-officer).

- An ordering/receiving procedure must be established with identified collecting points.

- Pregnant women are allowed to work with nanomaterials only with a special work authorization issued by an occupational physician.

- Lab safety audits are performed by occupational health and safety specialists.

- Permanent laboratory staff working in *Nano 2 *lab and every person working in *Nano 3 *are subject to medical surveillance On this question, reports [45] indicate that level of knowledge today doesn't allow proposing a specific medical survey, or indicators of exposure or effects. Still, certain consensus is obtained at international level [46] to recommend that potentially exposed workers should have periodical medical survey with 'conventional' exams, specific for potential target organ. One can think about respiratory tract or cardiovascular system. Results of these medical exams can also be source for data base to make epidemiological studies afterwards.

#### Personal measures

**Figure 7:**
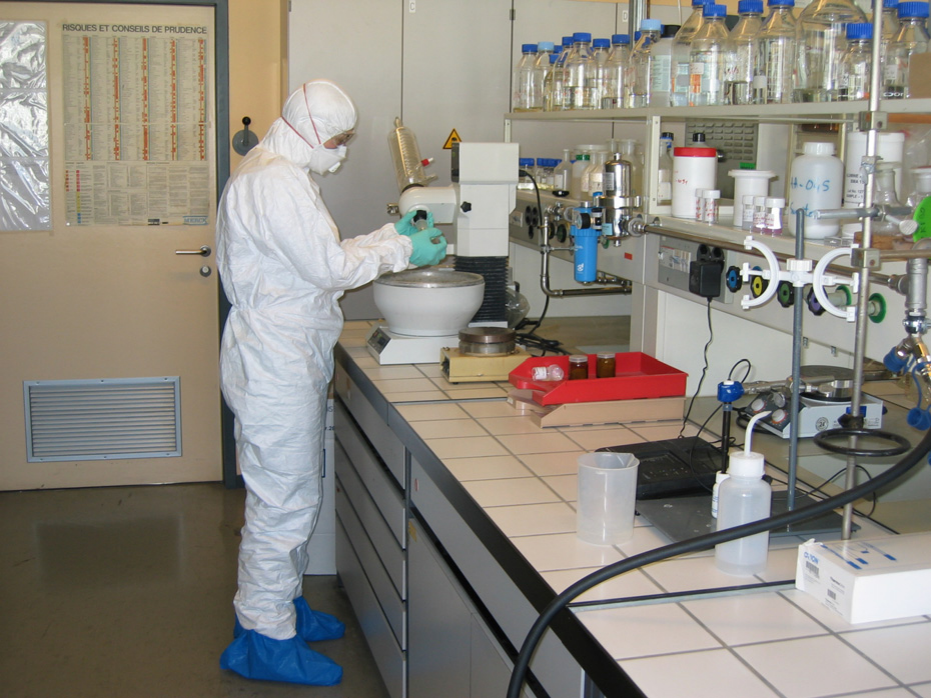
**Illustration of personal protective equipment for work with nano**. Illustration of personal equipment to be used in a Nano 3 laboratory.

Personal protective measures (see Figure 5 as well as Figure 7) assign specific equipments to different hazard levels. As example, a mask with Powered Air Respirator must be used if the work lasts over two hours (*Nano 3*), while P3 (EN 143) or FFP3 (EN 149)/P-100 (USA NIOSH) filter/filtering mask is accepted for shorter work periods. Protection of body parts depends on the hazard level. Two pairs of adapted protective gloves are mandatory when working in *Nano 2 *and above.

#### Cleaning management

Only *Nano 1 *laboratories can be cleaned by the regular (external) cleaning staff (see Figure 6) wearing protective equipment adapted to work in a chemical laboratory. *Nano 2 *must be cleaned by specially trained (external) personnel wearing the same protective equipment as lab employees and under the supervision of the lab responsible. *Nano 3 *must be cleaned exclusively by the lab employees themselves wearing the same personal protective equipment as for working and under supervision of a lab responsible. Only in exceptional cases, trained external personnel can be allowed to clean in *Nano 3 *laboratories under the supervision of the nano-officer.

## Conclusions

Present knowledge on nanomaterial toxicity is insufficient for completing precise risk assessment. Threshold Limit Values for nanomaterials do not exist nor is there standard equipment for sufficiently detailed routine exposure measurements. However, since preliminary scientific evaluations show that there are reasonable grounds for concern that activity with nanomaterials might have damaging effects on human health; the precautionary principle must be applied. Here we propose practical, clear and simple procedure for Nano safety and health management, which is a general approach based on the state of the nanomaterial in question (fibers and particles as powder, suspension, or in a solid matrix). New hazard knowledge will be used as it is developed and made available. The procedure proposes pragmatic mitigation measures that laboratories have to take for limiting exposures as much as considered reasonable. The lab responsible is in charge of applying measures adapted to specific activities. The proposed methodology and protective measures are provisional in nature pending the availability of more reliable scientific data. The procedure also allows estimating the number of research groups working in high hazard level laboratories. For reducing investment and operating cost, activities that classify laboratories into the highest hazard level should be centralized as much as possible. The methodology is being implemented at the Ecole Polytechnique de Lausanne in over 100 research labs dealing with nanomaterials; evaluation of its performance will be done when sufficient data are available.

## Competing interests

The authors declare that they have no competing interests.

## Authors' contributions

AG was in charge within the "nanosafe team" of the nano aspects related with physics. She contributed designing the decision tree and the safety measures matrix. She is the paper coordinator and main writer. APF helped designing the decision tree used for determination of laboratory (Nano hazard) type and helped writing the manuscript. She already runs a Nano3 laboratory and gave input on particle and suspension characteristics and characterizations. AM was involved in designing the decision tree used for determination of laboratory (Nano hazard) type and participated in the manuscript writing. He is in charge of a Nano3 laboratory at EPFL since 2003 and he gave input on particles, fibers and composites characteristics and characterizations. MR provided input about toxicological and exposure discussions, control banding knowledge and occupational hygiene strategies. He also supervised a Master study that investigated the situation of nano-workplaces at EPFL and which helped identify action domains. TM was the initiator and coordinator or the "nanosafe team" whose mission was to analyse nano activities in EPFL labs, to define strategies for laboratories classification and to implement adequate measures. He supervised a Master study on nano activities at EPFL that indentified potential hazards and led to the building of the "nanosafe team". All authors have read and approved the final manuscript.
